# Supplementary material for: A Critical View of the Application of the APEX Software (Aqueous Photochemistry of Environmentally-Occurring Xenobiotics) to Predict Photoreaction Kinetics in Surface Freshwaters
Source: Molecules. 2019 Dec 18;25(1):9. doi: 10.3390/molecules25010009 (PMC7017383; doi:10.3390/molecules25010009)
Supplement: Supplementary file 1 [file molecules-25-00009-s001.zip › 3.2.4_gcc-4.4.0.zip!/3.2.4_gcc-4.4.0/3.2.4_gcc-4.4.0/doc/octave/html/interpreter/Matrices.html]

Matrices - Untitled


Next: Ranges,
Up: Numeric Data Types


---

### 4.1 Matrices

It is easy to define a matrix of values in Octave. The size of the
matrix is determined automatically, so it is not necessary to explicitly
state the dimensions. The expression

```
     a = [1, 2; 3, 4]
```

results in the matrix

```
             /      \
             | 1  2 |
       a  =  |      |
             | 3  4 |
             \      /
```

Elements of a matrix may be arbitrary expressions, provided that the
dimensions all make sense when combining the various pieces. For
example, given the above matrix, the expression

```
     [ a, a ]
```

produces the matrix

```
     ans =
     
       1  2  1  2
       3  4  3  4
```

but the expression

```
     [ a, 1 ]
```

produces the error

```
     error: number of rows must match (1 != 2) near line 13, column 6
```

(assuming that this expression was entered as the first thing on line
13, of course).

Inside the square brackets that delimit a matrix expression, Octave
looks at the surrounding context to determine whether spaces and newline
characters should be converted into element and row separators, or
simply ignored, so an expression like

```
     a = [ 1 2
           3 4 ]
```

will work. However, some possible sources of confusion remain. For
example, in the expression

```
     [ 1 - 1 ]
```

the - is treated as a binary operator and the result is the
scalar 0, but in the expression

```
     [ 1 -1 ]
```

the - is treated as a unary operator and the result is the
vector `[ 1, -1 ]`. Similarly, the expression

```
     [ sin (pi) ]
```

will be parsed as

```
     [ sin, (pi) ]
```

and will result in an error since the `sin` function will be
called with no arguments. To get around this, you must omit the space
between `sin` and the opening parenthesis, or enclose the
expression in a set of parentheses:

```
     [ (sin (pi)) ]
```

Whitespace surrounding the single quote character (', used as a
transpose operator and for delimiting character strings) can also cause
confusion. Given `a = 1`, the expression

```
     [ 1 a' ]
```

results in the single quote character being treated as a
transpose operator and the result is the vector `[ 1, 1 ]`, but the
expression

```
     [ 1 a ' ]
```

produces the error message

```
     parse error:
     
       syntax error
     
     >>> [ 1 a ' ]
                   ^
```

because not doing so would cause trouble when parsing the valid expression

```
     [ a 'foo' ]
```

For clarity, it is probably best to always use commas and semicolons to
separate matrix elements and rows.

When you type a matrix or the name of a variable whose value is a
matrix, Octave responds by printing the matrix in with neatly aligned
rows and columns. If the rows of the matrix are too large to fit on the
screen, Octave splits the matrix and displays a header before each
section to indicate which columns are being displayed. You can use the
following variables to control the format of the output.

— Built-in Function: val = **output\_max\_field\_width** ()  
— Built-in Function: old\_val = **output\_max\_field\_width** (new\_val)  
> Query or set the internal variable that specifies the maximum width
> of a numeric output field.
>
>
>
> **See also:** format, output\_precision.

— Built-in Function: val = **output\_precision** ()  
— Built-in Function: old\_val = **output\_precision** (new\_val)  
> Query or set the internal variable that specifies the minimum number of
> significant figures to display for numeric output.
>
>
>
> **See also:** format, output\_max\_field\_width.

It is possible to achieve a wide range of output styles by using
different values of `output_precision` and
`output_max_field_width`. Reasonable combinations can be set using
the `format` function. See Basic Input and Output.

— Built-in Function: val = **split\_long\_rows** ()  
— Built-in Function: old\_val = **split\_long\_rows** (new\_val)  
> Query or set the internal variable that controls whether rows of a matrix
> may be split when displayed to a terminal window. If the rows are split,
> Octave will display the matrix in a series of smaller pieces, each of
> which can fit within the limits of your terminal width and each set of
> rows is labeled so that you can easily see which columns are currently
> being displayed. For example:
>
> ```
>           octave:13> rand (2,10)
>           ans =
>           
>            Columns 1 through 6:
>           
>             0.75883  0.93290  0.40064  0.43818  0.94958  0.16467
>             0.75697  0.51942  0.40031  0.61784  0.92309  0.40201
>           
>            Columns 7 through 10:
>           
>             0.90174  0.11854  0.72313  0.73326
>             0.44672  0.94303  0.56564  0.82150
> ```

Octave automatically switches to scientific notation when values become
very large or very small. This guarantees that you will see several
significant figures for every value in a matrix. If you would prefer to
see all values in a matrix printed in a fixed point format, you can set
the built-in variable `fixed_point_format` to a nonzero value. But
doing so is not recommended, because it can produce output that can
easily be misinterpreted.

— Built-in Function: val = **fixed\_point\_format** ()  
— Built-in Function: old\_val = **fixed\_point\_format** (new\_val)  
> Query or set the internal variable that controls whether Octave will
> use a scaled format to print matrix values such that the largest
> element may be written with a single leading digit with the scaling
> factor is printed on the first line of output. For example,
>
> ```
>           octave:1> logspace (1, 7, 5)'
>           ans =
>           
>             1.0e+07  *
>           
>             0.00000
>             0.00003
>             0.00100
>             0.03162
>             1.00000
> ```
>
> Notice that first value appears to be zero when it is actually 1. For
> this reason, you should be careful when setting
> `fixed_point_format` to a nonzero value.

- Empty Matrices
